# Acculturative stress, loneliness, smartphone addiction, L2 emotions, and creativity among international students in China: a structural equation model

**DOI:** 10.3389/fpsyt.2025.1585302

**Published:** 2025-05-26

**Authors:** Jian Gao, Dan Xu, Daniela Romano, Xuhui Hu

**Affiliations:** ^1^ School of International Education, Shantou University, Shantou, China; ^2^ Institute of Artificial Intelligence, De Montfort University, De Montfort, United Kingdom; ^3^ Department of Information studies, University College London, London, United Kingdom; ^4^ School of Chinese as a Second Language, Peking University, Beijing, China

**Keywords:** acculturative stress, loneliness, smartphone addiction, L2 learning emotions, creativity, structural equation model

## Abstract

**Introduction:**

International students in China often face psychological challenges such as acculturative stress, loneliness, and problematic smartphone use, which may affect their second language (L2) learning emotions and creativity. Although these factors have been studied individually, their interrelationships remain unclear.

**Methods:**

Data were collected from 213 international students studying in China using validated instruments: the *Acculturative* Stress Scale for International Students (ASSIS), *UCLA* Loneliness Scale, *Smartphone Addiction Scale Short Version* (SAS-SV), *Foreign Language Enjoyment and Classroom Anxiety Scale* (FLE & FLCA), and the *Inventory of Creative Activities and Achievements* (ICAA). Structural equation modeling (SEM) was employed to assess the hypothesized model and test both direct and indirect relationships among the constructs.

**Results:**

Acculturative stress significantly predicted smartphone addiction directly (*β* = 0.372, *p* < .001) and indirectly via loneliness (*β* = 0.169, *p* < .005). It also influenced FLCA through a chain mediation of loneliness and smartphone addiction (*β* = 0.135, *p* < .005). In terms of creativity, both acculturative stress (*β* = 0.300, *p* < .001) and FLE (*β* = 0.310, *p* < .001) positively predicted creative activities, which in turn strongly predicted creative achievement (*β* = 0.700, *p* < .001). FLCA was also positively related to creative achievement (*β* = 0.118, *p* = .016).

**Discussion:**

These findings support the Dual Pathway to Creativity Model and suggest that long-term moods (e.g., acculturative stress) and situational emotions (e.g., FLE and FLCA) may differentially affect creativity, aligning with the Hierarchical Model of Affect, Mood, and Emotion, advancing the understanding of international students' cross-cultural adaptation in the digital age.

## 1 Introduction

The landscape of international education has evolved significantly, with China emerging as a leading destination for global learners (1). Recent data from the State Council Information Office of China indicate that by September 2024, students from 195 countries and regions were pursuing education in China, with the total number of international Chinese language learners exceeding 200 million^1^. This educational internationalization presents unique challenges for students navigating cross-cultural environments, particularly in terms of acculturative stress and loneliness (2–6).

In this digital era, where global smartphone usage has reached 69.4% of the population (7) and Chinese university students average 6.21 hours of daily smartphone use (8), mobile devices serve dual roles as cognitive extensions (9) and potential sources of behavioral addiction (10–12).

Creativity, widely recognized as a cornerstone of contemporary education (13, 14), presents an intriguing paradox in relation to smartphone use and emotional states. While mobile devices can enhance divergent thinking through improved information access (15, 16) and boost critical thinking and creative self-efficacy (17), their excessive use may impair creativity by consuming cognitive resources and disrupting attentional processes (18–20). Similarly, the relationship between emotions and creativity remains complex, with some studies suggesting that negative emotions diminish cognitive flexibility (21, 22), while others argue that negative emotions can enhance persistence and effort, thus fostering creativity (23, 24).

While prior research has explored the individual impacts of acculturative stress, loneliness, smartphone addiction, and L2 emotions on international students (25–28), few studies have examined their combined interplay and collective influence on creativity, particularly in the context of international students in China. Moreover, the application of structural equation modeling (SEM) to investigate the mediating roles of loneliness, smartphone addiction, and L2 emotions in the relationship between acculturative stress and creativity remains underexplored. This study addresses these gaps by proposing and testing an SEM framework that elucidates these complex pathways, offering a novel contribution to understanding cross-cultural adaptation and creativity in a digital and multilingual educational setting.

### 1.1 Literature review

#### 1.1.1 Acculturative stress and loneliness in international students

International students constitute a distinct population that has attracted increasing scholarly attention in recent years (29). Unlike their domestic counterparts, these students face unique challenges stemming from their immersion in foreign, non-native language environments (30), which make them particularly susceptible to acculturative stress (3, 31). Acculturation is a multidimensional process in which individuals learn and adapt to a new culture while striving to maintain their original cultural identities. This process typically manifests through four primary strategies: assimilation, separation, marginalization, and integration (2, 3). Acculturative stress specifically refers to the psychological and social pressures individuals experience during the cultural adaptation process, which are often intensified by factors such as perceived discrimination and socioeconomic status (3, 32, 33). These pressures frequently manifest as heightened feelings of homesickness and loneliness among international students (34, 35).

Loneliness, defined as a distressing emotional experience arising from perceived deficiencies in social relationships (36), represents a significant adaptation challenge for this population (4, 37). Empirical studies have consistently documented elevated levels of loneliness among international students, with recent research focusing on the exacerbation of these feelings during the COVID-19 pandemic using both qualitative and quantitative methodologies (26, 38, 39).

Although extensive literature exists on the acculturation challenges faced by Chinese international students abroad (5, 6), comparatively fewer studies have focused on international students studying in China. As China has emerged as an increasingly popular destination for higher education, these students encounter unique cultural challenges that are distinct from those in traditional Western host countries. For instance, China’s high-context cultural framework - where communication relies heavily on implicit contextual cues, nonverbal signals, and shared cultural knowledge (40) - can amplify L2-related anxiety among students from low-context cultural backgrounds where communication tends to be more explicit and verbally direct (22, 41). A recent comparative study by Ngwira et al. revealed distinct patterns of acculturative stress among Asian and African international students in China, with African students reporting higher stress levels linked to perceived discrimination and Asian students identifying fear and guilt as primary stressors (25).

Moreover, China’s distinctive digital ecosystem shapes patterns of smartphone use, potentially creating additional adaptation obstacles (42, 43). Manu et al. found that international students in China strategically increased their social media usage to enhance cultural adaptation and campus engagement (44). Recent studies have also highlighted the challenges of homesickness and loss of social identity faced by international students in China, particularly during pandemic-related border closures (45, 46). These findings underscore the complex interplay among cultural adaptation, technological integration, and psychological well-being among international students in the Chinese educational context.

#### 1.1.2 Smartphone addiction

Unaddressed adaptation challenges may lead to problematic behaviors, such as a heightened risk of smartphone addiction (27, 47–49). Smartphone addiction is broadly defined as excessive and inappropriate reliance on smartphones to the extent that it interferes with daily life, social interactions, mental health, and academic or occupational performance (50, 51). This behavioral addiction manifests through several characteristic features: compulsive usage patterns (50), withdrawal symptoms when device access is restricted (52), tolerance development requiring increased usage to achieve the same psychological effects (53), and notable functional impairment across various life domains (54). Smartphone addiction shares common psychological and physiological mechanisms with other behavioral addictions, particularly in terms of reward system processing and impulse control (50, 55). Multiple studies have confirmed their adverse effects on student populations, including impaired sleep quality (56, 57), attention deficits (58, 59), deteriorated interpersonal relationships (60, 61), and compromised academic performance (62, 63). These findings were further substantiated by meta-analyses (10, 11, 64, 65).

Research on international students in China suggests that loneliness serves as a significant predictor of smartphone addiction (66, 67), with smartphones offering a ‘safer and less demanding’ medium for social interaction (68). Other psychological antecedents, including depression (69, 70) and social anxiety, have emerged as key predictors of problematic smartphone use (71–73). Notably, Elhai et al. introduced the concept of “Fear of Missing Out” (FoMO) (74), describing heightened dependence on smartphones as individuals seek to avoid missing critical social interactions or information. This phenomenon may be particularly relevant for international students attempting to maintain connections with their home culture and new social networks in their host countries.

#### 1.1.3 L2 emotions

Emotions constitute a central component of international students’ second language (L2) learning experiences (75, 76). Emotions in psychology are broadly defined as transient mental states characterized by physiological responses and observable behaviors (77). However, research in L2 learning contexts has predominantly emphasized negative emotional experiences (75, 78). In particular, anxiety has been identified as one of the most prominent emotional challenges in L2 learning and is conceptualized as a complex interplay of self-perceptions, beliefs, behaviors, and emotional reactions (79). Research has consistently demonstrated its inhibitory effects on L2 acquisition and performance (80).

More recently, however, the academic focus has pivoted toward investigating the role of positive emotions—aligned with the emerging field of positive psychology—in promoting effective L2 learning (81, 82). Among the positive emotions, foreign language enjoyment (FLE) has garnered significant attention. The FLE reflects the state in which psychological needs are met (83) and is considered a core element of positive psychology (84). Studies have consistently revealed that FLE positively correlates with students’ willingness to communicate (28), academic achievement (85), and reduced levels of L2 anxiety (86). The research community has developed specialized instruments to measure these L2-specific emotional states, such as the FLE scale and FLCA scale. While these instruments overlap with general emotion scales, they are specifically calibrated to capture the nuanced emotional dynamics of L2 classroom environments (75, 83). These tools provide insight into how emotions shape international students’ experiences and academic trajectories.

It is important to note that previous research may have conflated the concepts of mood and emotion (87). According to the Hierarchical Model of Affect, Mood, and Emotion proposed by Bianchi-Berthouze and Lisetti (88), mood (e.g., cheery and melancholy) typically persists for several days or longer, maintains a global focus and may not be attributed to a specific agency. In contrast, emotions (e.g., surprise, sadness, fear, anger) usually endure for only minutes, are tied to specific events or objects, and are triggered by either internal or external agency, with affect serving as an overarching concept encompassing both. In the context of international students, the stress and loneliness associated with their experiences likely represent long-lasting, persistent states (4, 37, 89), aligning more closely with the mood category. Conversely, short-term emotional experiences such as FLCA and FLE, which arise within specific contexts, align more closely with the definition of emotion (5, 6).

The relationship between smartphone use and emotional states demonstrates intricate, context-dependent patterns, as suggested by the Uses and Gratifications Theory (UGT) (90). The UGT posits that individuals turn to particular media forms, including smartphones, to fulfill various personal needs. In international students, feelings of loneliness and anxiety often drive increased smartphone dependence to satisfy the need for social connections and emotional regulation (67, 91). However, excessive smartphone use can paradoxically exacerbate social isolation and reduce engagement in physical activity (92), ultimately intensifying negative emotional states.

Classroom context adds further complexity to this relationship. In L2 learning scenarios, smartphone addiction has been linked to a diminished attention span, impaired language acquisition, and disrupted academic performance (47). Song et al. emphasized that overreliance on smartphones during L2 learning could weaken social support networks with peers and instructors, thereby increasing classroom anxiety in English as a Foreign Language (EFL) (93). Conversely, strategic smartphone use, such as utilizing mobile translation apps or grammar-check tools, has demonstrated the potential to reduce academic procrastination and enhance learners’ enjoyment of their L2 learning experiences (94).

Thus, the interplay between smartphone use and emotional state appears to be bidirectional (95). Although short-term adaptive smartphone use can alleviate anxiety and support productive study routines, long-term maladaptive patterns often lead to adverse emotional and academic outcomes. Distinguishing these contrasting effects remains a key challenge in the field, particularly in studies involving international student populations.

#### 1.1.4 Creativity

Creativity is recognized as a critical cognitive ability influencing individuals’ personal, academic, and professional development (96). Defined as the capacity to generate ideas, solutions, or products that are both original and useful (97), creativity encompasses diverse manifestations, ranging from everyday expressions (known as “little-c creativity”) to significant professional achievements (“Pro-C creativity”). Assessment approaches target either creative behaviors or creative outcomes. For instance, the Creative Behavior Inventory (CBI) (98) was designed to measure the frequency of creative behaviors, whereas the Creative Achievement Questionnaire (CAQ) (99) focused on assessing creative outcomes and accomplishments. The Inventory of Creative Activities and Achievements (ICAA) (100) successfully integrates both perspectives, measuring not only the frequency of creative activities but also levels of creative achievements. Unlike the CAQ, which emphasizes professional-level achievements (Pro-C, such as creative writing or scientific discoveries), the ICAA also includes creative accomplishments closer to everyday life (Little-C, such as sports or crafting).

In addition to measuring creative behaviors and achievements, divergent thinking tasks serve as widely used proxies for creativity. For instance, the Alternative Uses Task (AUT) (101, 102) asks participants to list alternative uses for a common object, with creativity scored along dimensions such as fluency (total number of responses), flexibility (diversity of categories represented by responses), and originality (uniqueness and quality of ideas). Another widely utilized tool is the Torrance Test of Creative Thinking (TTCT) (103), which assesses creativity through written and visual tasks, has been extensively adapted into multiple languages, and is employed globally (104, 105).

Furthermore, research has explored alternative dimensions of creativity, such as problem-solving creativity (106) and creative personality assessments, as exemplified by tools such as the Creative Personality Scale (CPS) (107) and more contemporary instruments (108). These multifaceted approaches underscore the inherent complexity of creativity and the contextual nuances that influence its manifestations.

These universal frameworks, however, require contextual adaptation when applied to populations navigating cross-cultural transitions. The investigation of creativity among international students offers unique academic value, given their exposure to diverse cultural environments. This multicultural experience fosters the integration of different perspectives (109), promoting cognitive complexity and flexibility in problem-solving (110). Research has consistently demonstrated that multicultural experiences and creative, supportive activities are positively correlated with innovative ideation (111), whereas emotional trust across cultural boundaries facilitates creative collaboration (112). From a language-learning perspective, bilinguals consistently demonstrate higher creativity levels than monolinguals (113, 114). This advantage stems from enhanced executive function developed through the management of multiple languages (115, 116). The use of a second language introduces unique emotional dynamics, with bilinguals often experiencing emotional distancing when using their L2, particularly negative emotional content (117, 118). Notably, recent research suggests that negative emotions have a diminished impact on creative performance in L2 settings compared to L1 environments, potentially enhancing bilinguals’ creative expression in their second language (119).

#### 1.1.5 Smartphone addiction and emotion as mediators between acculturative stress and creativity

Building upon these intercultural creative dynamics, the relationship between smartphone use and creativity exhibits complex and multifaceted patterns that warrant careful examination. Several studies have suggested that smartphone addiction impairs creativity by diverting attention, increasing cognitive load, and depleting the cognitive resources available for creative processes (18, 20, 120). By contrast, a substantial body of research has demonstrated that smartphone use may positively influence creativity by providing access to vast informational resources and diverse opportunities for social interaction, thereby fostering inspiration and ideation (15, 16). This positive influence is further supported by Guan et al., who found that smartphone use positively correlates with creative ideation through the mediating effects of critical thinking and creative self-efficacy (17). Additionally, mobile applications and interaction platforms may facilitate creativity by enhancing motivation, engagement, and collaboration (121, 122).

Recent meta-analytic findings by Olson et al. provided important nuance to this relationship, revealing that correlations between smartphone use and divergent thinking are generally small and inconsistent (rs = −0.09 to 0.09) (123). These inconsistencies suggest that contextual factors, particularly user personality traits, may moderate the relationship between smartphone use and creativity (124). Within this complex dynamic, emotion emerges as a critical mediator that influences the relationship between smartphone addiction and creativity (20, 125).

The emotional dimension becomes particularly salient when considering that smartphone addiction correlates with long-term emotional states, such as acculturative stress and loneliness, while short-term and situational emotional states play crucial roles in shaping creative potential (126). Positive emotions consistently enhance creativity by promoting cognitive flexibility and ideational originality (127–129). These positive emotional states activate memory retrieval (130) and broaden attentional scope (131), enabling individuals to approach problems more creatively (132).

The effects of negative emotions on creativity present a more nuanced perspective. Early research suggested that negative emotions could hinder creativity by reducing cognitive flexibility through approach-avoidance behaviors aimed at minimizing risks and errors (Regulatory Focus Theory) (21). Such avoidance tendencies may particularly impair willingness to engage in creative tasks (133–135). This perspective is supported by Baas et al.’s meta-analysis, spanning 25 years of research, which found that activating positive emotions correlated positively with creativity, whereas activating negative emotions showed negative correlations (128). In educational contexts, anxiety often leads students to adopt risk-avoidant behaviors and avoid challenging tasks that typically foster creative performance (136). Moreover, individual differences significantly influence this emotional-creativity relationship, with those possessing lower dispositional autonomy showing more pronounced effects of both positive and negative emotions on creativity (137).

However, emerging evidence suggests that negative emotions may enhance creativity under certain conditions. George and Zhou demonstrated that negative emotions can motivate individuals to exert additional effort and engage in more thorough problem-solving behaviors (23). Similarly, Roskes et al. found that negative emotions promote cognitive persistence and augment creative performance in tasks requiring sustained focus (138). This perspective is supported by Akinola and Mendes, who found that individuals with heightened biological vulnerability to stress exhibited enhanced artistic creativity in negative emotional states (139).

The Dual Pathway to Creativity Model (DPCM) proposed by Nijstad et al. offers a comprehensive framework for understanding these seemingly contradictory findings (24). This model posits that both positive and negative emotions can enhance creativity through distinct pathways; positive emotions predominantly foster cognitive flexibility, enabling divergent thinking, whereas negative emotions primarily promote cognitive persistence, facilitating a sustained focus on problem-solving tasks. Baas et al. extended this framework by examining personality traits and found that openness and other positive traits typically influence flexibility, whereas neuroticism and negative trait emotions drive persistence (140). Recent applications of DPCM across various contexts have provided robust empirical validation (141, 142).

Existing research has extensively examined acculturative stress, loneliness, smartphone addiction, L2 emotions, and creativity in isolation. However, few studies have investigated their combined dynamics, especially among international students in China. This study seeks to fill these gaps by exploring how these factors interact to influence creativity in this population.

### 1.2 The present study

This study investigates the complex interplay between acculturative stress and creativity among international students in China, employing a structural equation model (SEM) to test hypothesized relationships. Our approach is grounded in the Hierarchical Model of Affect, Mood, and Emotion (88), which provides a framework for distinguishing between long-term mood states and short-term emotions. Specifically, we classify acculturative stress and loneliness as enduring mood states that shape broader psychological tendencies over time. In contrast, L2 emotions—such as foreign language classroom anxiety (FLCA) and foreign language enjoyment (FLE)—are treated as transient, context-specific emotional responses tied to language learning experiences. Previous research on the connection between emotion and creativity has often induced short-term emotional states using videos or music (119), aligning with the definition of emotion rather than mood. This distinction is critical because enduring mood states like loneliness may establish a baseline influence on cognitive and behavioral tendencies (143), such as sustained engagement in creative activities, whereas transient emotions like FLCA and FLE may trigger immediate, context-specific responses that differentially shape creativity. International students in China are particularly susceptible to these dynamics due to cultural and language barriers (40, 41), restricted access to familiar digital platforms (42, 43), and academic pressures in a foreign educational environment (93, 94), amplifying the relevance of this model to their experiences.

Therefore, we propose a structural equation model that hypothesizes that acculturative stress and loneliness serve as contextual antecedents in a broader environment and influence creativity through the mediating effects of smartphone addiction and L2 learning emotions. Based on this framework, we tested the following hypotheses.

H1: Among international students in China, acculturative stress leads to smartphone addiction, with loneliness serving as a mediator.
H2: Acculturative stress affects L2 learning emotions (foreign language enjoyment and classroom anxiety), with loneliness and smartphone addiction serving as mediators.
H3: Acculturative stress influences creativity (creative activities and achievements) by mediating the effects of loneliness, smartphone addiction, and L2 learning emotions.

## 2 Research methods

### 2.1 Participants

Data were collected from October to November 2024 through an offline survey conducted at a university in China. The survey was administered via “WenJuanXing,” a professional Chinese survey platform. The questionnaire consisted of sections on informed consent, demographic information, and validated scales measuring acculturative stress, smartphone addiction, loneliness, and L2 learning emotions, as described in the next section.

Given the linguistic diversity of the international student population at this university, the questionnaire was provided in a primary English version as well as in three bilingual versions (English paired with Turkmen, Thai, or Russian). Since some scales did not have empirically validated versions in these languages and because the feasibility of machine translation has been verified (144)—we used ChatGPT to generate draft translations. Two native-speaking researchers specializing in linguistics and applied linguistics to each language cross-verified the drafts to ensure their reliability.

The survey distribution was facilitated through WeChat, China’s predominant social media platform, with instructors sharing survey invitations and links in international student classes. The inclusion criteria were as follows: (1) non-Chinese nationality; (2) current study experience in China; (3) age 18 years or older; (4) ownership and ability to use a personal smartphone; and (5) proficiency in reading English, Thai, Turkmen, or Russian. The survey took approximately 20 minutes to complete, and participants retained the right to withdraw or retract their data at any time during the process.

From the 222 completed questionnaires, we excluded six responses showing highly patterned answers and three containing logical inconsistencies (such as reporting extremely high creative achievement scores alongside minimal creative activity participation). The final analysis included data from 213 participants (107 males and 106 females), yielding an effective response rate of 95.95%. The sample comprised students from diverse native language backgrounds: Turkmen (n=102), Thai (n=34), Russian (n=45), and English (n=32). Participants represented various academic disciplines within the university. Due to the imbalance in group sizes across language backgrounds, the participants were analyzed as a collective group of international students rather than by individual language groups.

### 2.2 Measures

The survey collected demographic data, including age, sex, nationality, first language, study duration in China, and Chinese language proficiency. All measurement scales demonstrated robust reliability in our sample. Specific details are provided below.

*Acculturative Stress Scale for International Students (ASSIS)*

We used the ASSIS developed by Sandhu and Asrabadi to measure acculturative stress (145). This 36-item instrument encompasses seven dimensions: perceived discrimination, homesickness, perceived hate, fear, stress due to change, guilt, and non-specific miscellaneous concerns. Participants responded on a five-point Likert scale, with higher scores indicating greater perceived acculturative stress. The ASSIS has been validated extensively in studies on international students in China (146–148). The scale demonstrates excellent internal consistency (Cronbach’s alpha = 0.966).

*UCLA Loneliness Scale (Version 3)*

Loneliness was assessed using Version 3 of the UCLA Loneliness Scale, which comprises 20 items (149). Participants indicated the frequency of their experiences of loneliness on a four-point Likert scale. Total scores were calculated by adding the individual item responses, with higher scores reflecting increased loneliness levels. This scale has shown strong validity in previous studies on international students in China (66, 150). Our sample yielded high reliability (Cronbach’s α = 0.893).

*Smartphone Addiction Scale-Short Version (SAS-SV)*

Smartphone addiction was assessed using the short version of the Smartphone Addiction Scale (SAS-SV) (151), which has been validated in multiple empirical studies involving university students (152, 153) and in participants from different linguistic and cultural backgrounds (154–157). The SAS-SV consists of 10 items, with each item scored on a six-point Likert scale (1 = “strongly disagree” and 6: “strongly agree”). The final score was calculated by summing the scores of the ten selected items. Higher scores indicate a greater tendency toward smartphone addiction. In this study, Cronbach’s alpha for reliability was 0.842.

*L2 Learning Emotions Scales*

L2 learning emotions were measured using the Foreign Language Enjoyment Scale (FLE, 21 items) and the Foreign Language Classroom Anxiety Scale (FLCA, 8 items) developed by Dewaele and MacIntyre (83). Both scales employ five-point Likert responses, with mean scores calculated to reflect the levels of enjoyment and anxiety in L2 learning. These instruments have been widely validated among international students (158, 159). In our sample, both scales showed high reliability (FLE: Cronbach’s α = 0.815; FLCA: Cronbach’s α = 0.854).

*Inventory of Creative Activities and Achievements (ICAA)*

Creativity was assessed using the ICAA (100), which evaluates eight domains: Literature, Music, Arts and Crafts, Creative Cooking, Sports, Visual Arts (graphics, painting, cultivating, and architecture), Performing Arts (Theatre, dance, and film), and Science and Engineering. Each dimension was assessed for creative behavior and achievement. The Creative Activity (CAct) scale asks how frequently a participant has engaged in a particular activity in the past 10 years (from “never” to “more than 10 times”). The Creative Achievement (CAch) scale assesses creative achievement across 11 levels per domain (from “I have never been engaged in this domain” to “I have already sold some of my work in this domain”).

### 2.3 Analyses

Our analytical approach combined multiple statistical methods to examine the relationships among the study variables comprehensively. Initial analyses were conducted using SPSS 27 for descriptive statistics and Spearman’s correlation analyses. Subsequently, we employed structural equation modeling (SEM) using the lavaan package in R, with the maximum likelihood (ML) estimator, to test our hypothesized relationships. The ML estimator was selected given the robustness of our sample size and the continuous nature of the latent variables, though robust methods could be considered for Likert-scale data in future analyses.

The SEM framework incorporated acculturative stress as a predictor variable, with loneliness, smartphone addiction, and second language learning emotions (FLCA and FLE) serving as mediators that influenced creative activities and achievements. Gender was included as a control variable to account for potential demographic effects. To enhance model parsimony and robustness, we excluded non-significant paths with p > 0.20 from the final model. This threshold was chosen to retain paths with marginal significance (p < 0.20) that may hold theoretical relevance in this exploratory context, following recommendations for SEM in behavioral research (160). While this approach is more lenient than the conventional p > 0.05 cutoff, it ensures that potentially meaningful relationships are not prematurely discarded, though it may increase the risk of retaining weaker effects.

We employed two complementary approaches to ensure adequate statistical power and reliability. First, we conducted a *post hoc* power analysis using G*Power (version 3.1.9.4) based on the chi-squared value, sample size (N = 213), and effect size (w = 0.279). The analysis yielded a power level of 0.81 (1 - *β* error probability), exceeding the conventional threshold of 0.80. Additionally, we performed bootstrap analysis with 5,000 resamples to validate the stability of our parameter estimates. All key paths demonstrated 95% bias-corrected confidence intervals (excluding zero), confirming the reliability of our findings.

## 3 Results

Initial analyses included Spearman’s correlation to examine relationships among key variables, chosen over Pearson’s correlation due to its robustness to non-normal distributions and suitability for ordinal or non-linear data. The results revealed significant relationships among the study’s key variables, including Acculturative Stress (AS), loneliness (LON), Smartphone Addiction (SA), Foreign Language Enjoyment (FLE), Foreign Language Classroom Anxiety (FLCA), Creative Activity (CAct), and Creative Achievement (CAch). **Table 1** presents the correlations in detail.

**Table 1:** Spearman correlation coefficients between key variables.

Variable	LON	SA	FLE	FLCA	CAct	CAch
AS	.589** (<0.001)	.518** (<0.001)	-.240** (<0.001)	.594** (<0.001)	.206** (0.002)	.223** (0.001)
LON		.422** (<0.001)	-.276** (<0.001)	.503** (<0.001)	.196** (0.004)	.145* (0.035)
SA			-.116 (0.090)	.404** (<0.001)	.142* (0.038)	.094 (0.170)
FLE				-.192** (0.005)	.286** (<0.001)	.207** (0.002)
FLCA					.069 (0.315)	.192** (0.005)
CAct						.743** (<0.001)

Acculturative stress demonstrated a strong positive correlation with loneliness (r = .589, *p* <.001) and FLCA (r = .594, *p* <.001), along with a significant but weaker positive correlation with creative achievement (r = .223, *p* = .001). Notably, acculturative stress was negatively correlated with FLE (r = −.240, *p* <.001). These initial findings aligned with theoretical predictions and provided a foundation for more sophisticated structural equation modeling analyses. Descriptive statistics for the key variables are reported in **Appendix S1, Table S1**.

The structural equation model examining relationships among cultural adaptation stress, loneliness, smartphone use, emotional responses, and creativity demonstrated a good fit to the data: χ²(10) = 16.59, *p* = .084; CFI = 0.986, TLI = 0.962, RMSEA = 0.056 (90% CI: 0.000–0.101), SRMR = 0.035. The non-significant chi-square (*p* >.05) and excellent incremental fit indices (CFI/TLI > 0.95) supported the plausibility of the hypothesized structure.

**Figure 1:**
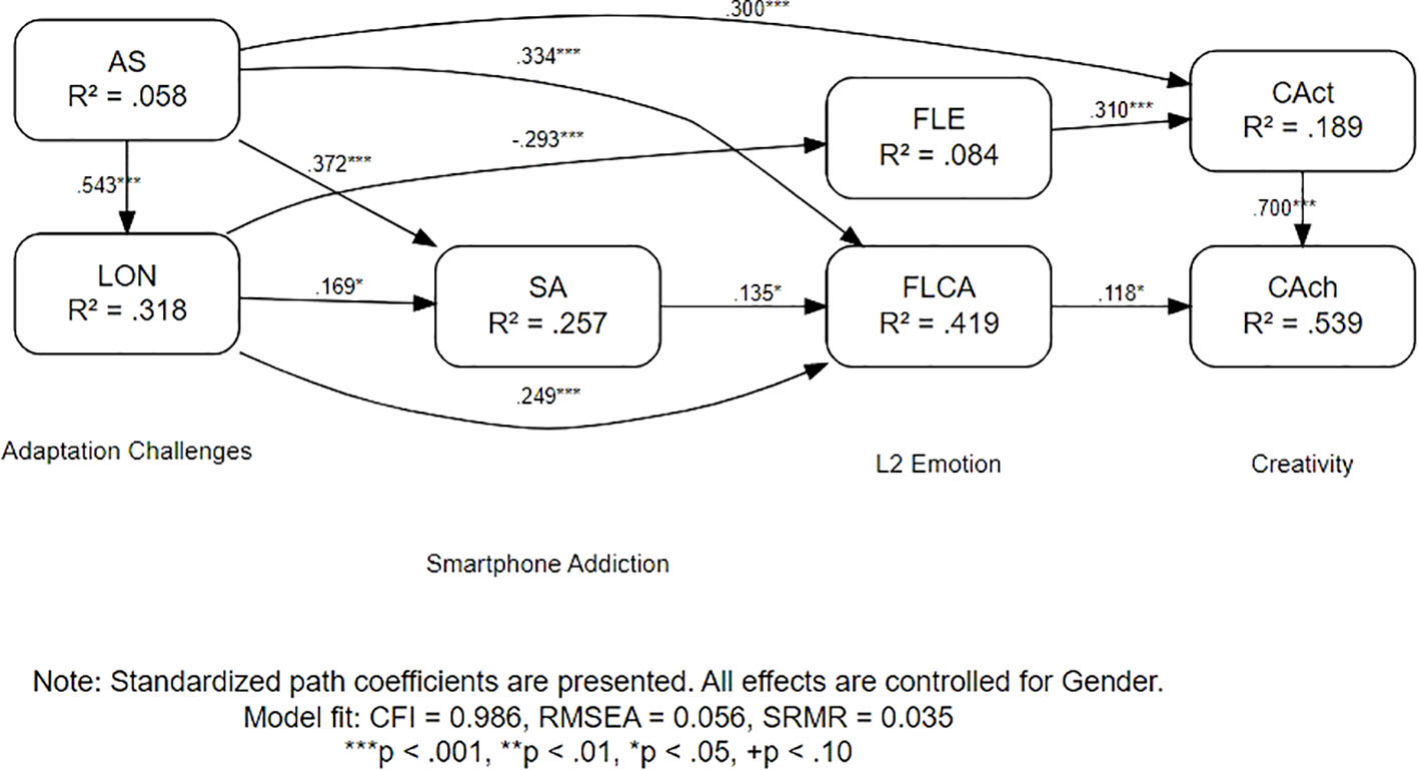
Path diagram of the structural equation model.

The standardized path coefficients revealed significant relationships (*p* <.05). Acculturative stress positively predicted loneliness (*β* = 0.543, 95% CI [4.713, 7.199], *p* <.001) and SA (*β* = 0.372, 95% CI [0.214, 0.529], *p* <.001). Loneliness, in turn, was associated with increased FLCA (*β* = 0.249, 95% CI [0.011, 0.035], *p* <.001) and reduced FLE (*β* = −0.293, 95% CI [−0.035, −0.004], *p* <.001). Acculturative stress showed a direct positive effect on FLCA (*β* = 0.135, 95% CI [0.008, 0.256], *p* = .026). Creative activity was significantly predicted by FLE (*β* = 0.31, 95% CI [0.195, 0.443], *p* <.001) and acculturative stress (*β* = 0.3, 95% CI [0.153, 0.456], *p* <.001), whereas creative achievement was influenced by creative activity (β = 0.7, 95% CI [0.586, 0.823], *p* <.001) and FLCA (*β* = 0.118, 95% CI [0.033, 0.210], *p* = .016). Sex exerted a significant negative effect on AS (*β* = −0.24, 95% CI [−0.738, −0.214], *p* <.001) and FLCA (*β* = −0.14, 95% CI [−0.320, −0.039], *p* = .008), but non-significant effects on other paths (*p* >.05). The model explained substantial variance in the key outcomes: 53.9% of creative achievement (*R*² = 0.539), 41.9% of FLCA (*R*² = 0.419), and 31.8% of loneliness (*R*² = 0.318). A path diagram is shown in **Figure 1**. The fully standardized path coefficients are detailed in **Supplementary Table S2**.

## 4 Discussion

This study employed structural equation modeling to elucidate the complex relationships between acculturative stress, loneliness, smartphone addiction, L2 learning emotions, and creativity among international students in China. Our findings validate and extend existing theoretical frameworks while offering novel insights into the interplay of these factors in the context of cross-cultural adaptation.

The results support Hypothesis 1 by demonstrating that international students experiencing acculturative stress are more susceptible to developing smartphone addiction, with loneliness serving as a mediating factor. Previous research in domestic contexts has identified loneliness as a key predictor of smartphone addiction (47, 49). The current study extends these findings to international students, revealing that acculturative stress exhibits a direct effect on smartphone addiction (*β* = 0.372, *p* <.001), accounting for 68.7% of its total predictive influence. Although the indirect pathway through loneliness remains statistically significant (*β* = 0.169, *p* <.005), the magnitude of the direct effect exceeds that of the indirect effect. This empirical pattern aligns with Ngwira et al.’s identification of cultural stressors as dominant predictors of behavioral maladaptation among international students (25, 161), highlighting the unique challenges faced by this population may fundamentally stems from cross-cultural adaptation challenges rather than generalized emotional distress.

These findings suggest that Berry’s traditional acculturation model may require refinement in the digital era (2, 3). Conventional models that heavily emphasize physical interactions, such as face-to-face socializing and community engagement (162), are being transformed by digital media. For instance, algorithm-driven “filter bubbles” (163) can confine users to familiar digital spaces, potentially hindering integration into local cultures. This dynamic may promote separation strategies and increase the reliance on smartphones to meet social needs (67, 91). These effects may be particularly pronounced in China’s rapidly evolving digital society (42, 43). Thus, our study highlights the importance of incorporating the influence of digital media into traditional adaptation frameworks and sets the stage for examining our second hypothesis regarding second language learning emotions.

Regarding Hypothesis 2, our results confirmed that acculturative stress influenced FLCA both directly (*β* = 0.334, *p* <.001) and indirectly through the mediating effects of loneliness and smartphone addiction (*β* = 0.135, *p* <.005). This finding aligns with the Hierarchical Model of Affect, Mood, and Emotion (87), suggesting that long-term emotional states (mood) such as acculturative stress and loneliness can affect short-term situational emotions through intermediary mechanisms such as smartphone addiction. According to Compensatory Internet Use Theory, individuals facing psychosocial difficulties—such as challenges in cultural adaptation—turn to digital media to address unmet emotional or social needs (164). For example, a student struggling with cultural isolation might use their smartphone to connect with friends or family in their home country through social media or messaging apps, seeking comfort and a sense of belonging. While this behavior may temporarily alleviate loneliness, it can paradoxically worsen FLCA by reducing opportunities for real-world interaction with the host culture and limiting L2 practice. Consequently, the student may feel less engaged in L2 settings, such as language classrooms, where proficiency is directly tested, thus heightening anxiety.

Cultural models of emotion further suggest that individualistic versus collectivistic backgrounds shape how emotions are conceptualized and experienced (165). Prolonged stress may alter the thalamocortical networks involved in emotion processing (166), potentially increasing the sensitivity to anxiety-inducing stimuli. In the context of language learning, acculturative stress can undermine self-confidence and self-efficacy (167), which, when combined with limited language proficiency, may create an inherent threat (79), further amplifying anxiety. These insights offer a new perspective that could help reconcile previous contradictory findings regarding the predictive roles of smartphone addiction and emotions (67, 91, 92). Future research should consider further differentiations between long-term mood states and short-term emotions.

Notably, our study found no significant direct correlation between acculturative stress and Foreign Language scores. While loneliness appeared to negatively affect FLE, its explanatory power remained limited (R² = 0.084), suggesting that neither acculturative stress nor loneliness were major predictors of FLE. This finding is consistent with Dewaele et al.’s framework, which posits that FLCA and FLE are distinct dimensions; students can experience both simultaneously rather than experiencing a simple seesaw relationship (75, 83, 168). Our research extends this framework by suggesting that long-term, diffuse negative moods, such as acculturative stress, may not directly impact FLE (169). Instead, FLE appears to be more strongly influenced by immediate factors, such as classroom activities and teacher support (84, 86).

These findings corroborate the Dual Pathway Model of Creativity (24). Both acculturative stress (*β* = 0.3, 95% CI [0.153, 0.456], *p* <.001) and FLE (*β* = 0.31, 95% CI [0.195, 0.443], *p* <.001) positively and significantly predicted a higher frequency of creative activity with moderate effect sizes. In turn, creative activity mediates the influence on creative achievement (*β* = 0.7, 95% CI [0.586, 0.823], *p* <.001), echoing previous literature that highlights the positive role of positive emotions on creativity (127, 128, 132).

A particularly noteworthy finding is that FLCA showed no direct association with creative activity but positively predicted creative achievement (*β* = 0.118, 95% CI [0.033, 0.210], *p* = .016). This finding underscores the need to distinguish between long- and short-term emotions in the creative process. Although both acculturative stress and FLCA are viewed as negative affective states, their mechanisms of influence on creativity appear to differ, which might explain the inconsistent findings regarding the impact of negative emotions on creativity (128).

In cross-cultural settings, prolonged negative mood may prompt students to engage more deeply with the local culture (44), including increased participation in creative activities. This engagement often activates alternative creative strategies, particularly evident in cross-cultural language practices such as sign language communication (170, 171). Furthermore, acculturative stress appears to be linked to intercultural sensitivity, with highly sensitive individuals demonstrating an enhanced ability to integrate cultural differences and mitigate the negative impact of stress on creativity through effective emotional regulation (172, 173).

Conversely, anxiety, as a situational emotion, appears to operate through different mechanisms, potentially leading individuals to enhance the quality of their creative output through iterative refinements (24, 138) rather than simply increasing the frequency of creative activities. This distinction in creative outcomes may also reflect the differences in measurement approaches. While previous research has often focused on divergent thinking (e.g., using an alternative use task) or overall creativity assessments (e.g., the Torrance Tests of Creative Thinking) (104, 105, 174), our study specifically measured both creative activities and achievements, providing a more comprehensive view of how different emotional states influence various aspects of creative performance.

## 5 Conclusion and future research

This study advances our understanding of how cross-cultural acculturative stress, loneliness, smartphone addiction, second language learning emotions, and creativity interrelate among international students in the digital age. Our findings confirmed that acculturative stress exerts indirect effects through the mediating roles of loneliness and smartphone addiction, highlighting its distinctive impact on the behavioral and emotional states of international students. The results provide strong support for the Dual Pathway to Creativity Model, demonstrating that both acculturative stress and foreign language enjoyment can positively contribute to creative activities. Furthermore, our findings validate the Hierarchical Model of Affect, Mood, and Emotion in explaining the complex interplay between negative emotions and creativity. Particularly in cross-cultural environments, enduring emotional states such as acculturative stress may propel students to engage more proactively in creative endeavors, whereas anxiety can enhance creative output quality through iterative refinement processes.

This study has several limitations that warrant consideration and suggest directions for future research. First, the reliance on self-report measures may introduce a subjective bias. Future investigations should incorporate diverse data collection methods, including physiological assessments and behavioral observations, to achieve methodological triangulation and strengthen the reliability of the findings. Second, because our sample was limited to international students in China, the generalizability of our findings to different cultural contexts remains uncertain. Students from individualistic versus collectivist backgrounds may exhibit distinct adaptation patterns, necessitating future research that broadens the sample diversity and facilitates multidimensional comparisons of cross-cultural adaptation processes.

Additionally, while our study delineated the relationships between acculturative stress, second language learning emotions, and creativity, it did not fully address the temporal dynamics of these associations. Given that international students’ adaptation states likely evolve over time, longitudinal research designs would be valuable in examining trends in acculturative stress, loneliness, and smartphone addiction, as well as their long-term effects on learning emotions and creative performance.

Finally, while this study emphasized the application of the Hierarchical Model of Affect, Mood, and Emotion, its efficacy in cross-cultural settings requires further empirical validation. Future research should explore the interactions among various emotional types during acculturation adaptation and assess how these distinct emotions specifically influence academic and creative development. The potential moderating role of cross-cultural sensitivity on the relationship between acculturative stress and creativity merits further investigation. Subsequent studies could focus on elucidating the underlying mechanisms through which cultural differences mediated by emotion regulation affect students’ creative output.

Future research should strive to refine and deepen our understanding of the interconnections between cross-cultural acculturative stress, emotional experiences, and creativity in international students by employing diverse data sources, enhancing sample heterogeneity, adopting longitudinal approaches, and extending the current theoretical frameworks. These efforts will contribute to more effective support systems for international students and enhance our understanding of creative development in cross-cultural educational contexts.

## Data Availability

The original contributions presented in the study are included in the article/**Supplementary Material**. Further inquiries can be directed to the corresponding author/s.

## Footnotes

1 The state council information office of the people’s republic of China. Press Con. (2024). https://www.scio.gov.cn/live/2024/34857/index_m.html.

## References

[1] Cao C Meng Q. A systematic review of predictors of international students’ cross-cultural adjustment in China: Current knowledge and agenda for future research. Asia Pac Educ Re. (2022) 23:45–67. doi: 10.1007/s12564-021-09700-1

[2] Berry JW. Conceptual approaches to understanding acculturation. In: Chun KM Organista PB Marín G, editors. Acculturation: Advances in theory, measurement, and applied researc. Washington DC, USA: American Psychological Association (2003), 17–38. doi: 10.1037/10472-004

[3] Berry JW. Stress perspective on acculturation. In: Sam D Berry JW, editors. Handbook of acculturation psycholog. Cambridge, United Kingdom: Cambridge University Press (2006), 43–57. doi: 10.1017/CBO9780511489891.007

[4] Alasmari AA. Challenges and social adaptation of international students in Saudi Arabia. Heliyo. (2023) 9:e16283. doi: 10.1016/j.heliyon.2023.e16283 PMC10208833 37234614

[5] Su S Lin X McElwain A. Parenting, loneliness, and stress in Chinese international students: do parents still matter from thousands of miles away? J Fam Stu. (2023) 29:255–68. doi: 10.1080/13229400.2021.1910540

[6] Bilecen B Diekmann I Faist T. Loneliness among Chinese international and local students in Germany: the role of student status, gender, and emotional support. Eur J High Edu. (2024) 14:470–88. doi: 10.1080/21568235.2023.2215992

[7] DataReportal. Digital 2023: Global overview report(2024). Available online at: https://datareportal.com/reports/digital-2024-global-overview-report/ (Accessed April 10, 2025).

[8] Li L Niu Z Griffiths MD Mei S. The smartphone addiction scale: Psychometric properties, invariance, network perspective, and latent profile analysis among a sample of Chinese university students. Int J Ment Health Addic. (2024) 22:24–46. doi: 10.1007/s11469-022-00857-3

[9] Smart P. Extended cognition and the Internet: A review of current issues and controversies. Philos Techn. (2017) 30:357–90. doi: 10.1007/s13347-016-0250-2 PMC6961510 32010552

[10] Chen G Lyu C. The relationship between smartphone addiction and procrastination among students: A systematic review and meta-analysis. Pers Individ Diff. (2024) 224:112652. doi: 10.1016/j.paid.2024.112652

[11] Leow MQH Chiang J Chua TJX Wang S Tan NC. The relationship between smartphone addiction and sleep among medical students: A systematic review and meta-analysis. PloS On. (2023) 18:290724. doi: 10.1371/journal.pone.0290724 PMC10503710 37713408

[12] Mendez ML Padrón I Fumero A Marrero RJ. Effects of internet and smartphone addiction on cognitive control in adolescents and young adults: A systematic review of fMRI studies. Neurosci Biobehav Re. (2024) 159:105572. doi: 10.1016/j.neubiorev.2024.105572 38320657

[13] Henriksen D Henderson M Creely E Ceretkova S Černochová M Sendova E. Creativity and technology in education: An international perspective. Technol Knowl Lear. (2018) 23:409–24. doi: 10.1007/s10758-018-9380-1

[14] Li Y Kim M Palkar J. Using emerging technologies to promote creativity in education: A systematic review. Int J Educ Res Ope. (2022) 3:100177. doi: 10.1016/j.ijedro.2022.100177

[15] Singh MKK Samah NA. Impact of smartphone: A review on positive and negative effects on students. Asian Soc Sc. (2018) 14:83. doi: 10.5539/ass.v14n11p83

[16] Chung S Lee KY Choi J. Exploring digital creativity in the workspace: The role of enterprise mobile applications on perceived job performance and creativity. Comput Hum Beha. (2015) 42:93–109. doi: 10.1016/j.chb.2014.03.055

[17] Guan J Yang Y Ma W Li G Liu C. The relationship between mobile phone use and creative ideation among college students: The roles of critical thinking and creative self-efficacy. Psychol Aesthet Crea. (2024). doi: 10.1037/aca0000695

[18] Wilmer HH Sherman LE Chein JM. Smartphones and cognition: A review of research exploring the links between mobile technology habits and cognitive functioning. Front Psycho. (2017) 8:605. doi: 10.3389/fpsyg.2017.00605 PMC5403814 28487665

[19] Hadar A Hadas I Lazarovits A Alyagon U Eliraz D Zangen A. Answering the missed call: Initial exploration of cognitive and electrophysiological changes associated with smartphone use and abuse. PloS On. (2017) 12:e0180094. doi: 10.1371/journal.pone.0180094 PMC5497985 28678870

[20] Xu C Tang C Huo L Wang R Wang Y Liu C. How does technology overload impair creativity performance? The mediating roles of emotion and state self-control. Mediat Roles Emot State Self-Contro. (2023). doi: 10.2139/ssrn.4425618

[21] Idson LC Liberman N Higgins ET. Distinguishing gains from nonlosses and losses from nongains: A regulatory focus perspective on hedonic intensity. J Exp Soc Psycho. (2000) 36:252–74. doi: 10.1006/jesp.1999.1402

[22] Chiu FC Hsu CC Lin YN Liu CH Chen HC Lin CH. Effects of creative thinking and its personality determinants on negative emotion regulation. Psychol Re. (2019) 122:916–43. doi: 10.1177/0033294118775973 29860928

[23] George JM Zhou J. Understanding when bad moods foster creativity and good ones don’t: the role of context and clarity of feelings. J Appl Psycho. (2002) 87:687. doi: 10.1037/0021-9010.87.4.687 12184573

[24] Nijstad BA Dreu CK Rietzschel EF Baas M. The dual pathway to creativity model: Creative ideation as a function of flexibility and persistence. Eur Rev Soc Psycho. (2010) 21:34–77. doi: 10.1080/10463281003765323

[25] Ngwira FF Adamseged HY Kondowe W. Acculturative stress and socio-demographic influences: A comparative study of Asian and African international students in China. J Intercult Commu. (2024) 24:191–9. doi: 10.36923/jicc.v24i4.990

[26] Phillips R Seaborne K Goldsmith A Curtis N Davies A Haynes W. Student loneliness through the pandemic: How, why and where? Geogr. (2022) 188:277–93. doi: 10.1111/geoj.12438 PMC9111728 35600138

[27] Ahmed AH Elemo AS Hamed OAO. Smartphone addiction and phubbing in international students in Turkey: the moderating role of mindfulness. J Coll Stud De. (2023) 64:64–78. doi: 10.1353/csd.2023.0002

[28] Lee JS Xie Q Lee K. Informal digital learning of English and L2 willingness to communicate: Roles of emotions, gender, and educational stage. J Multiling Multicult De. (2024) 45:596–612. doi: 10.1080/01434632.2021.1918699

[29] Jin R Wei J Yin M Le TT. Navigating between worlds: Cultural identity and emigration intentions of chinese international student returnees in the post-COVID era. Curr Psycho. (2024) 43:1–12. doi: 10.1007/s12144-024-06774-z

[30] Gatwiri G. The influence of language difficulties on the wellbeing of international students: An interpretive phenomenological analysis. Inq. (2015) 7. Available online at: http://www.inquiriesjournal.com/a?id=1042 (Accessed April 10, 2025).

[31] Poyrazli S. Psychological symptoms and concerns experienced by international students: Outreach implications for counseling centers. J Int Stu. (2014) 5:306–12. doi: 10.32674/jis.v5i3.424

[32] Beiser MN Hou F. Ethnic identity, resettlement stress and depressive affect among Southeast Asian refugees in Canada. Soc Sci Me. (2006) 63:137–50. doi: 10.1016/j.socscimed.2005.12.002 16436309

[33] Yamada M. The role of social presence in learner-centered communicative language learning using synchronous computer-mediated communication: Experimental study. Comput Edu. (2009) 52:820–33. doi: 10.1016/j.compedu.2008.12.007

[34] Cao C Zhu C Meng Q. A survey of the influencing factors for international academic mobility of Chinese university students. High Educ. (2016) 70:200–20. doi: 10.1111/hequ.12084

[35] Wawera AS McCamley A. Loneliness among international students in the UK. J Furth High Edu. (2020) 44:1262–74. doi: 10.1080/1097198X.2023.2166752

[36] Perlman D Peplau LA. Toward a social psychology of loneliness. Pers Relatsh. (1981) 3:31–56.

[37] Wu HP Garza E Guzman N. International student’s challenge and adjustment to college. Educ Res In. (2015) 2015:202753. doi: 10.1155/2015/202753

[38] Maleku A Kim YK Kirsch J Um MY Haran H Yu M. The hidden minority: Discrimination and mental health among international students in the US during the COVID-19 pandemic. Health Soc Care Community. (2022) 30:2419–32. doi: 10.1111/hsc.13683 34921449

[39] Chang LC Dattilo J Huang FH. Relationships of leisure social support and flow with loneliness in international students in Taiwan: Implications during the COVID-19 pandemic. Leis Sc. (2024) 46:1013–30. doi: 10.1080/01490400.2022.2056550

[40] Hall ET. Beyond Culture. New York, NY: Doubleday. (1976).

[41] Zhu LY Bauman CW Young MJ. Unlocking creative potential: Reappraising emotional events facilitates creativity for conventional thinkers. Organ Behav Hum Decis Process. (2023) 174:104209. doi: 10.1016/j.obhdp.2022.104209

[42] Wang X Alauddin M Zafar AU Zhang Q Ahsan T Barua Z. WeChat Moments among international students: Building Guanxi networks in China. J Glob Inf Technol Manag. (2023) 26:47–76. doi: 10.1080/1097198X.2023.2166752

[43] Cao C Meng Q Zhang H. A longitudinal examination of WeChat usage intensity, behavioral engagement, and cross-cultural adjustment among international students in China. High Edu. (2024) 87:661–83. doi: 10.1007/s10734-023-01029-5 PMC10057683 37362755

[44] Dwumah Manu B Ying F Oduro D Antwi J Yakubu Adjuik R. The impact of social media use on student engagement and acculturative stress among international students in China. PloS On. (2023) 18:284185. doi: 10.1371/journal.pone.0284185 PMC10104301 37058453

[45] Mekonen YK Adarkwah MA. Exploring homesickness among international students in China during border closure. Int J Intercult Rela. (2023) 94:101800. doi: 10.1016/j.ijintrel.2023.101800 PMC10029356 36968191

[46] Raja R Ma J Zhang M Li XY Almutairi NS Almutairi AH. Social identity loss and reverse culture shock: Experiences of international students in China during the COVID-19 pandemic. Front Psycho. (2023) 14:994411. doi: 10.3389/fpsyg.2023.994411 PMC9948652 36844319

[47] Karaoglan Yilmaz FG Avci U Yilmaz R. The role of loneliness and aggression on smartphone addiction among university students. Curr Psycho. (2023) 42:17909–17. doi: 10.1007/s12144-022-03018-w PMC8933130 35340690

[48] Gan Y Zhang T Zhang J Wu X Shao M. Impact of mobile game addiction tendency on Chinese university students: A hierarchical linear modeling study. Front Psycho. (2022) 13:937446. doi: 10.3389/fpsyg.2022.937446 PMC9291722 35859844

[49] Sönmez M Gürlek Kısacık Ö Eraydın C. Correlation between smartphone addiction and loneliness levels in nursing students. Perspect Psychiatr Car. (2021) 57:82–7. doi: 10.1111/ppc.12527 32424870

[50] Griffiths M. Behavioural addiction: an issue for everybody? Empl Counc Toda. (1996) 8:19–25. doi: 10.1108/13665629610116872

[51] Shambare R Rugimbana R Zhowa T. Are mobile phones the 21st century addiction? Afr J Bus Mana. (2012) 6:573. doi: 10.5897/AJBM11.1940

[52] Billieux J Maurage P Lopez-Fernandez O Kuss DJ Griffiths MD. Can disordered mobile phone use be considered a behavioral addiction? An update on current evidence and a comprehensive model for future research. Curr Addict Re. (2015) 2:156–62. doi: 10.1007/s40429-015-0054-y

[53] Kwon M Lee JY Won WY Park JW Min JA Hahn C. Development and validation of a smartphone addiction scale (SAS. PloS On. (2013) 8:56936. doi: 10.1371/journal.pone.0056936 PMC3584150 23468893

[54] Lin Y Liu Y Fan W Tuunainen VK Deng S. Revisiting the relationship between smartphone use and academic performance: A large-scale study. Comput Hum Beha. (2021) 122:106835. doi: 10.1016/j.chb.2021.106835

[55] Busch PA McCarthy S. Antecedents and consequences of problematic smartphone use: A systematic literature review of an emerging research area. Comput Hum Beha. (2021) 114:106414. doi: 10.1016/j.chb.2020.106414

[56] Demirci K Akgönül M Akpinar A. Relationship of smartphone use severity with sleep quality, depression, and anxiety in university students. J Behav Addict. (2015) 4:85–92. doi: 10.1556/2006.4.2015.010 26132913 PMC4500888

[57] Nikolic A Bukurov B Kocic I Vukovic M Ladjevic N Vrhovac M. Smartphone addiction, sleep quality, depression, anxiety, and stress among medical students. Front Public Healt. (2023) 11:1252371. doi: 10.3389/fpubh.2023.1252371 PMC10512032 37744504

[58] Park Y Lee S. Gender differences in smartphone addiction and depression among Korean adolescents: Focusing on the internal mechanisms of attention deficit and self-control. Comput Hum Beha. (2022) 136:107400. doi: 10.1016/j.chb.2022.107400

[59] Kwon SJ Kim Y Kwak Y. Influence of smartphone addiction and poor sleep quality on attention-deficit hyperactivity disorder symptoms in university students: A cross-sectional study. J Am Coll Healt. (2022) 70:209–15. doi: 10.1371/journal.pone.0056936 32240033

[60] Seo DG Park Y Kim MK Park J. Mobile phone dependency and its impacts on adolescents’ social and academic behaviors. Comput Hum Beha. (2016) 63:282–92. doi: 10.1016/j.chb.2016.05.026

[61] Hong FY Lin CC Lin TJ Huang DH. The relationship among the social norms of college students, and their interpersonal relationships, smartphone use, and smartphone addiction. Behav Inf Techno. (2021) 40:415–26. doi: 10.1080/0144929X.2019.1699959

[62] Samaha M Hawi NS. Relationships among smartphone addiction, stress, academic performance, and satisfaction with life. Comput Hum Beha. (2016) 57:321–5. doi: 10.1016/j.chb.2015.12.045

[63] Alotaibi MS Fox M Coman R Ratan ZA Hosseinzadeh H. Smartphone addiction prevalence and its association on academic performance, physical health, and mental well-being among university students in Umm Al-Qura University (UQU. Saudi Arab Int J Environ Res Public Healt. (2022) 19:3710. doi: 10.3390/ijerph19063710 PMC8954621 35329397

[64] Yang J Fu X Liao X Li Y. Association of problematic smartphone use with poor sleep quality, depression, and anxiety: A systematic review and meta-analysis. Psychiatry Re. (2020) 284:112686. doi: 10.1016/j.psychres.2019.112686 31757638

[65] Zhong Y Ma H Liang YF Liao CJ Zhang CC Jiang WJ. Prevalence of smartphone addiction among Asian medical students: A meta-analysis of multinational observational studies. Int J Soc Psychiatr. (2022) 68:1171–83. doi: 10.1177/00207640221089535 35422151

[66] Jiang Q Li Y Shypenka V. Loneliness, individualism, and smartphone addiction among international students in China. Cyberpsychology Behav Soc Netw. (2018) 21:711–8. doi: 10.1089/cyber.2018.0115 30328694

[67] Mehmood A Bu T Zhao E Zelenina V Alexander N Wang W. Exploration of psychological mechanism of smartphone addiction among international students of China by selecting the framework of the I-PACE model. Front Psycho. (2021) 12:758610. doi: 10.3389/fpsyg.2021.758610 PMC8632695 34867657

[68] Sánchez-Martínez M Otero A. Factors associated with cell phone use in adolescents in the community of Madrid (Spain. Cyberpsychol Beha. (2009) 12:131–7. doi: 10.1089/cpb.2008.0164 19072078

[69] Kim MO Kim H Kim K Ju S Choi J Yu MI. Smartphone addiction:(focused depression, aggression and impulsion) among college students. Indian J Sci Techno. (2015) 8:1–6. doi: 10.17485/ijst/2015/v8i25/80215

[70] Matar Boumosleh J Jaalouk D. Depression, anxiety, and smartphone addiction in university students-A cross sectional study. PloS On. (2017) 12:182239. doi: 10.1371/journal.pone.0182239 PMC5544206 28777828

[71] Enez Darcin A Kose S Noyan CO Nurmedov S Yılmaz O Dilbaz N. Smartphone addiction and its relationship with social anxiety and loneliness. Behav Inf Techno. (2016) 35:520–5. doi: 10.1080/0144929X.2016.1158319

[72] Turgeman L Hefner I Bazon M Yehoshua O Weinstein A. Studies on the relationship between social anxiety and excessive smartphone use and on the effects of abstinence and sensation seeking on excessive smartphone use. Int J Environ Res Public Healt. (2020) 17:1262. doi: 10.3390/ijerph17041262 PMC7068314 32075336

[73] Khan S Atta M Malik NI Makhdoom IF. Prevalence and relationship of smartphone addiction, nomophobia, and social anxiety among college and university late adolescents. Ilk Online. (2021) 20:3588–95. doi: 10.17051/ilkonline.2021.05.394

[74] Elhai JD Yang H Montag C. Fear of missing out (FOMO): overview, theoretical underpinnings, and literature review on relations with severity of negative affectivity and problematic technology use. Braz J Psychiatr. (2020) 43:203–9. doi: 10.1016/j.addbeh.2019.106261 PMC8023172 32401865

[75] Dewaele JM Li C. Emotions in second language acquisition: A critical review and research agenda. Foreign Lang World. (2020) 196:34–49. Available online at: https://eprints.bbk.ac.uk/id/eprint/32797 (Accessed April 10, 2025).

[76] Hu X Gao J Romano D. Exploring the effects of multiple online interaction on emotions of L2 learners in synchronous online classes. Heliyon. (2024) 10:e37619. doi: 10.1016/j.heliyon.2024.e37619 39309791 PMC11416288

[77] Ekman P. An argument for basic emotions. Cognit Emo. (1992) 6:169–200. doi: 10.1080/02699939208411068

[78] Jo H Baek EM. Exploring the dynamics of mobile app addiction: the interplay of communication, affective factors, flow, perceived enjoyment, and habit. BMC Psycho. (2023) 11:404. doi: 10.1186/s40359-023-01440-8 PMC10662456 37986198

[79] Horwitz EK Horwitz MB Cope J. Foreign language classroom anxiety. Mod Lang. (1986) 70:125–32. doi: 10.1111/j.1540-4781.1986.tb05256.x

[80] MacIntyre PD Vincze L. Positive and negative emotions underlie motivation for L2 learning. Stud Second Lang Learn Teach. (2017) 7:61–88. doi: 10.14746/ssllt.2017.7.1.4

[81] Li C Wei L. Anxiety, enjoyment, and boredom in language learning amongst junior secondary students in rural China: How do they contribute to L2 achievement? Stud Second Lang Acqui. (2023) 45:93–108. doi: 10.1017/S0272263122000031

[82] Solhi M Shirvan ME Benlioğlu B. Enjoyment begets enjoyment: An experience sampling study on the impact of L2 teacher enjoyment on EFL learners’ learning enjoyment and willingness to communicate. System. (2024) 126:103493. doi: 10.1016/j.system.2024.103493

[83] Dewaele JM MacIntyre PD. The two faces of Janus? Anxiety and enjoyment in the foreign language classroom. Stud Second Lang Learn Teach. (2014) 4:237–74. doi: 10.14746/ssllt.2014.4.2.5

[84] Li C Jiang G Dewaele JM. Understanding Chinese high school students’ foreign language enjoyment: validation of the Chinese version of the foreign language enjoyment scale. System. (2018) 76:183–96. doi: 10.1016/j.system.2018.06.004

[85] Dewaele JM Li C. Foreign language enjoyment and anxiety: Associations with general and domain-specific English achievement. Chin J Appl Linguist. (2022) 45:32–48. doi: 10.1515/CJAL-2022-0104

[86] Dewaele JM Witney J Saito K Dewaele L. Foreign language enjoyment and anxiety: The effect of teacher and learner variables. Lang Teach Re. (2018) 22:676–97. doi: 10.1177/1362168817692161

[87] Beedie C Terry PC Lane AN. Distinctions between emotion and mood. Cognit Emot. (2005) 19:847–78. doi: 10.1080/02699930541000057

[88] Bianchi-Berthouze N Lisetti CL. Modeling multimodal expression of user’s affective subjective experience. User Model User-Adapt Interac. (2002) 12:49–84. doi: 10.1023/a:1013365332180

[89] Qualter P Vanhalst J Harris R Roekel E Lodder G Bangee M. Loneliness across the life span. Perspect Psychol Sc. (2015) 10:250–64. doi: 10.1177/1745691615568999 25910393

[90] Blumler JG. The role of theory in uses and gratifications studies. Commun Re. (1979) 6:9–36. doi: 10.1177/009365027900600102

[91] Contractor AA Weiss NH Elhai JD. Examination of the relation between PTSD symptoms, smartphone feature uses, and problematic smartphone use. Soc Sci Comput Re. (2019) 37:385–403. doi: 10.1177/0894439318770745

[92] Dimidjian S Barrera M Martell C Muñoz RF Lewinsohn PM. The origins and current status of behavioral activation treatments for depression. Annu Rev Clin Psycho. (2011) 7:1–38. doi: 10.1146/annurev-clinpsy-032210-104535 21275642

[93] Song Y Sznajder K Bai Q Xu Y Dong Y Yang X. English as a foreign language writing anxiety and its relationship with self-esteem and mobile phone addiction among Chinese medical students—A structural equation model analysis. PloS On. (2023) 18:284335. doi: 10.1371/journal.pone.0284335 PMC10118181 37079547

[94] Corbita DP. Direct and indirect effects of academic procrastination, academic emotions and use of smartphone on EFL writing competency among arabic speakers. In: Innovative and Intelligent Digital Technologies; Towards an Increased Efficienc. vol. p. Springer Nature Switzerland, Cham (2024). p. 207–15. doi: 10.1007/978-3-031-70399-7_15

[95] Lu X Katoh T Chen Z Nagata T Kitamura T. Text messaging: Are dependency and excessive use discretely different for Japanese university students? Psychiatry Re. (2014) 216:255–62. doi: 10.1016/j.psychres.2013.12.024 24560613

[96] Amabile TM. Creativity In Context: Update To The Social Psychology Of Creativity (1st ed.). New York, USA: Routledge. (1996). doi: 10.4324/9780429501234

[97] Runco MA Jaeger GJ. The standard definition of creativity. Creat Res. (2012) 24:92–6. doi: 10.1080/10400419.2012.650092

[98] Hocevar D. (1979). The development of the creative behavior inventory, in: Paper presented at the annual meeting of the Rocky Mountain Psychological Association. (ERIC Document Reproduction Service No. Ed. 170 350).

[99] Carson SH Peterson JB Higgins DM. Reliability, validity, and factor structure of the creative achievement questionnaire. Creat Res. (2005) 17:37–50. doi: 10.1207/s15326934crj1701_4

[100] Diedrich J Jauk E Silvia PJ Gredlein JM Neubauer AC Benedek M. Assessment of real-life creativity: The Inventory of Creative Activities and Achievements (ICAA. Psychol Aesthet Creat Arts. (2018) 12:304–16. doi: 10.1037/aca0000137

[101] Guilford JP. The nature of human intelligenc. New York, NY: McGraw-Hill (1967).

[102] Reiter-Palmon R Forthmann B Barbot B. Scoring divergent thinking tests: A review and systematic framework. Psychol Aesthet Creat Arts. (2019) 13:144. doi: 10.1037/aca0000227.supp

[103] Torrance EP. Torrance tests of creative thinking: Norms technical manual (Research Edition). Princeton, USA: Personnel Press. (1966).

[104] Alabbasi AMA Paek SH Kim D Cramond B. What do educators need to know about the Torrance Tests of Creative Thinking: A comprehensive review. Front Psycho. (2022) 13:1000385. doi: 10.3389/fpsyg.2022.1000385 PMC9644186 36389550

[105] Xu X Chen Y Ye X Zhang J Luan J Li Y. Creativity: exploring factor structure of the torrance tests of creative thinking in Chinese preschoolers. J Creat Beha. (2024), 1–15. doi: 10.1002/jocb.1529

[106] Treffinger DJ Isaksen SG Stead-Dorval KB. Creative problem solving: An introductio. New York, USA: Routledge (2023).

[107] Gough HG. A creative personality scale for the adjective check list. J Pers Soc Psycho. (1979) 37:1398. doi: 10.1037/0022-3514.37.8.1398

[108] Ayyildiz P Yilmaz A. Moving the Kaleidoscope’to see the effect of creative personality traits on creative thinking dispositions of preservice teachers: The mediating effect of creative learning environments and teachers’ creativity fostering behavior. Think Ski Creat. (2021) 41:100879. doi: 10.1016/j.tsc.2021.100879

[109] Benet-Martínez V Lee F Leu J. Biculturalism and cognitive complexity: Expertise in cultural representations. J Cross-Cult Psycho. (2006) 37:386–407. doi: 10.1177/0022022106288476

[110] Chiu C Hong Y. Cultural competence: Dynamic processes. In: Elliot A Dweck CS, editors. Handbook of motivation and competenc. Guilford, New York (2005). p. 489–505.

[111] Leung AKY Chiu CY. Multicultural experience, idea receptiveness, and creativity. J Cross-Cult Psycho. (2010) 41:723–41. doi: 10.1177/0022022110361707

[112] Chua RY Morris MW Mor S. Collaborating across cultures: Cultural metacognition and affect-based trust in creative collaboration. Organ Behav Hum Decis Process. (2012) 118:116–31. doi: 10.1016/j.obhdp.2012.03.009

[113] Lambert WE Tucker GR d’Anglejan A. Cognitive and attitudinal consequences of bilingual schooling: The St. Lambert project through grade five. J Educ Psycho. (1973) 65:141–59. doi: 10.1037/h0034983

[114] Kharkhurin AV. Bilingual verbal and nonverbal creative behavior. Int J Biling. (2010) 14:211–26. doi: 10.1177/1367006910363060

[115] Rodriguez-Fornells A Diego Balaguer R Münte TF. Executive control in bilingual language processing. Lang Lear. (2006) 56:133–90. doi: 10.1111/j.1467-9922.2006.00359.x

[116] Zabelina DL O’Leary D Pornpattananangkul N Nusslock R Beeman M. Creativity and sensory gating indexed by the P50: Selective versus leaky sensory gating in divergent thinkers and creative achievers. Neuropsychologia. (2015) 69:77–84. doi: 10.1016/j.neuropsychologia.2015.01.034 25623426

[117] Jończyk R Boutonnet B Musiał K Hoemann K Thierry G. The bilingual brain turns a blind eye to negative statements in the second language. Cognit Affect Behav Neurosc. (2016) 16:527–40. doi: 10.3758/s13415-016-0411-x PMC4868866 26926623

[118] Zhang W Jończyk R Wu YJ Lan Y Gao Z Hu J. Brain potentials reveal how emotion filters native language access when bilinguals read words in their second language. Cereb Cortex. (2023) 33:8783–91. doi: 10.1093/cercor/bhad161 37160328

[119] Jończyk R Naranowicz M Dębowska-Kozłowska K Bromberek-Dyzman K. Negative mood constrains creative thinking in the native but not in the second language. Think Ski Creat. (2024) 51:101457. doi: 10.1016/j.tsc.2023.101457

[120] Upshaw JD Davis WM Zabelina DL. iCreate: Social media use, divergent thinking, and real-life creative achievement. Transl Issues Psychol Sc. (2022) 8:125. doi: 10.1037/tps0000306

[121] Tang C Mao S Naumann SE Xing Z. Improving student creativity through digital technology products: A literature review. Think Ski Creat. (2022) 44:101032. doi: 10.1016/j.tsc.2022.101032

[122] Booton SA Kolancali P Murphy VA. Touchscreen apps for child creativity: An evaluation of creativity apps designed for young children. Comput Edu. (2023) 201:104811. doi: 10.1016/j.compedu.2023.104811

[123] Olson JA Sandra DA Langer EJ Raz A Veissière SP. Creativity and smartphone use: Three correlational studies. Int J Human–Computer Interac. (2023) 39:2920–5. doi: 10.1080/10447318.2022.2088451

[124] Cocoradă E Maican CI Cazan AM Maican MA. Assessing the smartphone addiction risk and its associations with personality traits among adolescents. Child Youth Serv Re. (2018) 93:345–54. doi: 10.1016/j.childyouth.2018.08.006

[125] Brailovskaia J Margraf J. The relationship between active and passive Facebook use, Facebook flow, depression symptoms and Facebook Addiction: a three-month investigation. J Affect Disord Re. (2022) 10:100374. doi: 10.1016/j.jadr.2022.100374

[126] Celume MP Ivcevic Z Zenasni F. Mood and creativity in children: Differential impacts on convergent and divergent thinking. Psychol Aesthet Crea. (2023). doi: 10.1037/aca0000577

[127] Ashby FG Isen AM. A neuropsychological theory of positive affect and its influence on cognition. Psychol Re. (1999) 106:529. doi: 10.1037/0033-295x.106.3.529 10467897

[128] Baas M Dreu CK Nijstad BA. A meta-analysis of 25 years of mood-creativity research: Hedonic tone, activation, or regulatory focus? Psychol Bul. (2008) 134:779–806. doi: 10.1037/a0012815.supp 18954157

[129] Davis MA. Understanding the relationship between mood and creativity: A meta-analysis. Organ Behav Hum Decis Process. (2009) 108:25–38. doi: 10.1016/j.obhdp.2008.04.001

[130] Isen AM. Positive affect and decision making M. In: Lewis JMH, editor. Handbook of emotion. Guilford Press, New York (1993). p. 261–77.

[131] Fredrickson BL. The role of positive emotions in positive psychology: The broaden-and-build theory of positive emotions. Am Psycho. (2001) 56:218. doi: 10.1037/0003-066X.56.3.218 PMC3122271 11315248

[132] Kumar M Roy S Bhushan B Sameer A. Creative problem solving and facial expressions: a stage based comparison. PloS On. (2022) 17:e0269504. doi: 10.1371/journal.pone.0269504 PMC9216609 35731723

[133] Brockner J Higgins ET. Regulatory focus theory: Implications for the study of emotions at work. Organ Behav Hum Decis Process. (2001) 86:35–66. doi: 10.1006/obhd.2001.2972

[134] Runco MA Chand I. Cognition and creativity. Educ Psychol Re. (1995) 7:243–67. doi: 10.1007/BF02213373

[135] Carver CS. Negative affects deriving from the behavioral approach system. Emotion. (2004) 4:3–22. doi: 10.1037/1528-3542.4.1.3 15053723

[136] Ivcevic Z Hoffmann JD. The creativity dare: Attitudes toward creativity and prediction of creative behavior in school. J Creat Beha. (2022) 56:239–57. doi: 10.1002/jocb.527

[137] Xiao F Wang L Chen Y Zheng Z Chen W. Dispositional and situational autonomy as moderators of mood and creativity. Creat Res. (2015) 27:76–86. doi: 10.1080/10400419.2015.992683

[138] Roskes M Dreu CK Nijstad BA. Necessity is the mother of invention: avoidance motivation stimulates creativity through cognitive effort. J Pers Soc Psycho. (2012) 103:242. doi: 10.1037/a0028442 22564013

[139] Akinola M Mendes WB. The dark side of creativity: Biological vulnerability and negative emotions lead to greater artistic creativity. Pers Soc Psychol Bul. (2008) 34:1677–86. doi: 10.1177/0146167208323933 PMC2659536 18832338

[140] Baas M Roskes M Sligte D Nijstad BA Dreu CK. Personality and creativity: The dual pathway to creativity model and a research agenda. Soc Pers Psychol Compass. (2013) 7:732–48. doi: 10.1111/spc3.12062

[141] Nijstad BA Rietzschel EF Baas M Dreu CK. The dual pathway to creativity model: Implications for workplace creativity. In: Handbook of research on creativity and innovatio. Cheltenham, United Kingdom: Edward Elgar Publishing (2021). p. 28–48. doi: 10.4337/9781788977272.00010

[142] Baas M. In the mood for creativity. In: SternbergEds JCKJ, editor. The Cambridge handbook of creativit. Cambridge, United Kingdom: Cambridge University Press (2019), 257–72. doi: 10.1017/9781316979839.014

[143] Hawkley LC. Loneliness matters: A theoretical and empirical review of consequences and mechanisms. Ann Behav Me. (2010) 40:218–27. doi: 10.1007/s12160-010-9210-8 PMC3874845 20652462

[144] Son J Kim B. Translation performance from the user’s perspective of large language models and neural machine translation systems. Information. (2023) 14:574. doi: 10.3390/info14100574

[145] Sandhu DS Asrabadi BR. Development of an acculturative stress scale for international students: Preliminary findings. Psychol Re. (1994) 75:435–48. doi: 10.2466/pr0.1994.75.1.435 7809315

[146] Yu B Chen X Li S Liu Y Jacques-Tiura AJ Yan H. Acculturative stress and influential factors among international students in China: A structural dynamic perspective. PloS On. (2014) 9:96322. doi: 10.1371/journal.pone.0096322 PMC4005751 24788357

[147] Liu Y Chen X Li S Yu B Wang Y Yan H. Path analysis of acculturative stress components and their relationship with depression among international students in China. Stress Healt. (2016) 32:524–32. doi: 10.1002/smi.2658 26762565

[148] Shan C Hussain M Sargani GR. A mix-method investigation on acculturative stress among Pakistani students in China. PloS On. (2020) 15:240103. doi: 10.1371/journal.pone.0240103 PMC7531799 33007007

[149] Russell DW. UCLA Loneliness Scale (Version 3): Reliability, validity, and factor structure. J Pers Assess. (1996) 66:20–40. doi: 10.1207/s15327752jpa6601_2 8576833

[150] Jiang Q Yuen M Horta H. Factors influencing life satisfaction of international students in Mainland China. Int J Adv Couns. (2020) 42:393–413. doi: 10.1007/s10447-020-09409-7 32836578 PMC7398760

[151] Kwon M Kim DJ Cho H Yang S. The smartphone addiction scale: development and validation of a short version for adolescents. PloS On. (2013) 8:83558. doi: 10.1371/journal.pone.0083558 PMC3877074 24391787

[152] Kaya F Bostanci Daştan N Durar E. Smartphone usage, sleep quality, and depression in university students. Int J Soc Psychiatr. (2021) 67:407–14. doi: 10.1177/0020764020960207 32969293

[153] Kumcagiz H Gündüz Y. Relationship between psychological well-being and smartphone addiction in university students. Int J High Edu. (2016) 5:144–56. doi: 10.5430/ijhe.v5n4p144

[154] Akın A Altundağ Y Turan ME Akın U. The validity and reliability of the Turkish version of the smart phone addiction scale-short form for adolescent. Procedia-Soc Behav Sc. (2014) 152:74–7. doi: 10.1016/j.sbspro.2014.09.157

[155] Haug S Castro RP Kwon M Filler A Kowatsch T Schaub MP. Smartphone use and smartphone addiction among young people in Switzerland. J Behav Addict. (2015) 4:299–307. doi: 10.1556/2006.4.2015.037 26690625 PMC4712764

[156] Arthy CC Effendy E Amin MM Loebis B Camellia V Husada MS. Indonesian version of addiction rating scale of smartphone usage adapted from smartphone addiction scale-short version (SAS-SV) in junior high school. Open Access Maced J Med Sc. (2019) 7:3235–9. doi: 10.3889/oamjms.2019.691 PMC6953939 31949522

[157] Andrade ALM Scatena A Martins GDG Oliveira Pinheiro B Silva AB Enes CC. Validation of smartphone addiction scale–Short version (SAS-SV) in Brazilian adolescents. Addict Beha. (2020) 110:106540. doi: 10.1016/j.addbeh.2020.106540 32682269

[158] Qi D Haladin NAB. The relationship between positive psychology health and language competence of Chinese international students: the role of foreign language enjoyment. Arch Clin Psychiatr. (2022) 49. doi: 10.15761/0101-60830000000447

[159] Sun J. (2022). Foreign language anxiety and achievement: A case study of prospective overseas high schoolers in China, in: 2022 International Conference on Science Education and Art Appreciation (SEAA 2022). Amsterdam, Netherlands: Atlantis Press, 1106–12. doi: 10.2991/978-2-494069-05-3_133

[160] Kline RB. Principles and practice of structural equation modelin. New York, USA: Guilford publications (2023).

[161] Wu Y Liu W Liu A Lin-Schilstra L Lyu P. International students’ mental health care in China: A systematic review. Healthcare. (2021) 9:1634. doi: 10.3390/healthcare9121634 34946359 PMC8700832

[162] Verile MG Ertl MM Dillon FR Rosa M. Acculturative stress among Latina young adult immigrants: The mediating role of receiving community context. Transl Issues Psychol Sc. (2019) 5:91. doi: 10.1037/tps0000185 PMC12312122 40746496

[163] Nguyen TT Hui PM Harper FM Terveen L Konstan JA. (2014). Exploring the filter bubble: the effect of using recommender systems on content diversity, in: Proceedings of the 23rd international conference on World wide web. New York, NY: Association for Computing Machinery, 677–86. doi: 10.1145/2566486.2568012

[164] Kardefelt-Winther D. A conceptual and methodological critique of internet addiction research: Towards a model of compensatory internet use. Comput Hum Beha. (2014) 31:351–4. doi: 10.1016/j.chb.2013.10.059

[165] Mesquita B Markus HR. Culture and emotion: the cultural construction of emotional experience. Annu Rev Psycho. (2019) 70:67–93. doi: 10.1016/j.neubiorev.2024.105572

[166] Wager TD Kang J Johnson TD Nichols TE Satpute AB Barrett LF. A Bayesian model of category-specific emotional brain responses. PloS Comput Bio. (2015) 11:1004066. doi: 10.1371/journal.pcbi.1004066 PMC4390279 25853490

[167] Du H Li X Lin D Tam CC. Collectivistic orientation, acculturative stress, cultural self-efficacy, and depression: A longitudinal study among Chinese internal migrants. Community Ment Health. (2015) 51:239–48. doi: 10.1007/s10597-014-9785-9 PMC4297733 25480108

[168] Dewaele JM MacIntyre PD. Do flow, enjoyment and anxiety emerge equally in English foreign language classrooms as in other foreign language classrooms? Rev Bras Lingüíst Ap. (2022) 22:156–80. doi: 10.1590/1984-6398202218487

[169] Proietti Ergün AL Ersöz Demirdağ H. The relation between Foreign Language Enjoyment, subjective well-being, and perceived stress in multilingual students. J Multiling Multicult De. (2024) 45:2575–87. doi: 10.1080/01434632.2022.2057504

[170] McLay L. Hands-on learning: The influence of hand gestures on children’s recall of scientific informatio. New Zealand: Open Access Te Herenga Waka-Victoria University of Wellington (2017).

[171] Frith E Miller SE. Creativity in motion: examining the impact of meaningful movement on creative cognition. Front Cogn. (2024) 3:1386375. doi: 10.3389/fcogn.2024.1386375 PMC13281090 42339450

[172] He J Song X Wang C Zhang R. Intercultural sensitivity as a mediator in the relationship between implicit intercultural identification and emotional disturbance—An exploratory study of international high school students. Front Psychiatr. (2023) 14:1098671. doi: 10.3389/fpsyt.2023.1098671 PMC10175589 37187861

[173] Ching GS Chao PC Lien WC. Acculturative hassles and strategies: Relationship between study abroad related depression, anxiety, and stress. Int J Psychol Stu. (2014) 3:3–25. doi: 10.5861/ijrsp.2014.818

[174] Said-Metwaly S Noortgate W Barbot B. Torrance test of creative thinking-verbal, Arabic version: Measurement invariance and latent mean differences across gender, year of study, and academic major. Think Ski Creat. (2021) 39:100768. doi: 10.1016/j.tsc.2020.100768

