# Supplementary material for: Acculturative stress, loneliness, smartphone addiction, L2 emotions, and creativity among international students in China: a structural equation model
Source: Front Psychiatry. 2025 May 26;16:1585302. doi: 10.3389/fpsyt.2025.1585302 (PMC12147449; doi:10.3389/fpsyt.2025.1585302)
Supplement: Supplementary file 1 [file Table1.docx]

### **Appendix S1. Descriptive Statistics for Key Variables**

**Table S1. Descriptive Statistics for Key Variables**

| **Variable** | **N** | **Minimum** | **Maximum** | **Mean** | **Std. Error** | **Std. Deviation** |
| --- | --- | --- | --- | --- | --- | --- |
| Smartphone Addiction (SA) | 213 | 10 | 54 | 25.4789 | 0.66869 | 9.75916 |
| Loneliness (LON) | 213 | 20 | 72 | 40.5869 | 0.7499 | 10.94447 |
| Foreign Language Enjoyment (FLE) | 213 | 2.57 | 4.9 | 4.0362 | 0.02994 | 0.43691 |
| Foreign Language Classroom Anxiety (FLCA) | 213 | 1 | 5 | 2.8903 | 0.06273 | 0.91546 |
| Acculturative Stress (AS) | 213 | 36 | 159 | 70.4038 | 1.88079 | 27.4492 |
| Creative Activity (CAct) | 213 | 0 | 29.67 | 7.4421 | 0.40871 | 5.96494 |
| Creative Achievement (CAch) | 213 | 0 | 186 | 34.9577 | 2.71559 | 39.63267 |

### **Appendix** **S2. Full Parameter Estimates**

Table S2. Full Parameter Estimates

| **Path** | **β** | **SE** | **z-value** | **p-value** | **95% CI** |
| --- | --- | --- | --- | --- | --- |
| AS ← Gender | -0.24 | 0.133 | -3.615 | <.001 | [-0.738, -0.214] |
| LON ← AS | 0.543 | 0.638 | 9.317 | <.001 | [4.713, 7.199] |
| SA ← AS | 0.372 | 0.072 | 5.148 | <.001 | [0.214, 0.529] |
| SA ← LON | 0.169 | 0.007 | 2.36 | 0.018 | [0.002, 0.029] |
| FLCA ← SA | 0.135 | 0.061 | 2.23 | 0.026 | [0.008, 0.256] |
| FLCA ← AS | 0.334 | 0.068 | 4.932 | <.001 | [0.199, 0.476] |
| FLCA ← LON | 0.249 | 0.006 | 3.881 | <.001 | [0.011, 0.035] |
| FLE ← LON | -0.293 | 0.006 | -4.373 | <.001 | [-0.035, -0.004] |
| Cact ← FLE | 0.31 | 0.063 | 4.957 | <.001 | [0.195, 0.443] |
| Cact ← AS | 0.3 | 0.065 | 4.657 | <.001 | [0.153, 0.456] |
| Cach ← Cact | 0.7 | 0.048 | 14.67 | <.001 | [0.586, 0.823] |
| Cach ← FLCA | 0.118 | 0.05 | 2.405 | 0.016 | [0.033, 0.210] |
